# The Use of Patient Reported Outcome Measures (PROMs) 6 Months Post-Stroke and Their Association with the National Institute of Health Stroke Scale (NIHSS) on Admission to Hospital

**DOI:** 10.3390/geriatrics6030088

**Published:** 2021-09-07

**Authors:** Jonathan Hewitt, Natalie Bains, Katherine Wallis, Stephanie Gething, Anna Pennington, Ben Carter

**Affiliations:** 1 Division of Population Medicine, Cardiff University, Cardiff CF10 3AT, UK; bainsnm@cardiff.ac.uk (N.B.); wallisk@cardiff.ac.uk (K.W.); gethings@cardiff.ac.uk (S.G.); anna.pennington@wales.nhs.uk (A.P.); 2 Department of Biostatistics and Health Informatics, Institute of Psychiatry, Psychology & Neuroscience, King’s College London, London WC2R 2LS, UK; ben.carter@kcl.ac.uk

**Keywords:** patient reported outcome measures, PROMs, stroke

## Abstract

Patient Reported Outcome Measures (PROMs) assess clinical outcomes from the perspective of the patient. The stroke community recommended fifteen questions for use in stroke survivors, based on the established PROMIS10 with five additional stroke-specific questions. This study aimed to determine its association with the National Institute of Health Stroke Scale (NIHSS) on admission. PROM responses were taken from an existing randomised control trial and, using secondary analysis, the total score was calculated out of 100. The association between PROMs and NIHSS was estimated. Using a multivariable regression, an adjusted mean difference (aMD) in PROM total score for the baseline clinical characteristics was calculated. 343 participants (16.3%) completed the PROM; mean age 71.7 (30–94) years; 133 women (38.8%). There was a strong association between increasing NIHSS Scores on admission to hospital and worsening PROM scores at 6 months (*p* = 0.002). There was consistency between the NIHSS and modified Rankin score with the stroke-specific domain and total PROM scores. When adjusted, women had lower (worse) total PROM scores, with aMD = −3.85 (95% CI −6.30–−1.41; *p* = 0.002) and so did haemorrhagic strokes, with a reduction of 3.88 (95% CI −0.61–7.37; *p* = 0.097). This study contributes to the evaluation process of this stroke-specific PROM and emphasises that stroke severity on admission correlates with poorer patient outcomes 6 months following a stroke, especially in women and those suffering haemorrhagic stroke.

## 1. Introduction

Patient reported outcome measures (PROMs) are standardised and validated assessments which consider clinical outcomes from the perspective of the patient, rather than the health care provider. They are becoming increasingly common and focus on a patient’s personal view of their health following an illness or intervention.

Numerous generic PROMs are available, ranging in length and complexity. For example, the widely used EQ5D, which is a short, simple and validated measure, or the longer SF36, which is based on eight different health domains [1,2]. Many generic PROMs have been used in stroke survivors, but they lack specific relevance to stroke survivors and the clinical conditions they suffer [3].

To help address this problem, Salinas et al. (2016) developed a patient reported outcome measure for use in stroke survivors [4]. This PROMs, developed together by clinicians and stroke survivors, consists of fifteen questions. Ten questions were taken from the PROMIS-10, an established PROM with a mental and physical health domain. An additional five stroke-specific questions were developed by the International Consortium for Health Outcomes Measurement (ICHOM) [4,5], however, this questionnaire requires validation.

The aims of the study were to determine if there is an association between the NIHSS on admission to hospital and the stroke-specific 15 Patient Reported Outcome Measure (PROM) questions, asked six months following a stroke. Secondary outcomes included validating the two domains of the PROMIS10 questionnaire and one stroke-specific domain, as well as to determine demographic predictors of worsening severity on the PROM.

## 2. Materials and Methods

### 2.1. Trial Design

Patients receive a post-diagnosis 6-month review as part of their routine care. Responses from 15 patient reported outcome measures (PROMs) were collected from stroke survivors in this review, in a Zelen’s design non-inferiority randomised controlled trial of four follow up assessment methods; face-to face, telephone, online or by post [6]. The full protocol and results to assess the follow up rates are available [6,7]. Patient demographics, clinical characteristics and health-related data were collected on admission; this included the classification of stroke (ischaemic or haemorrhagic) as defined by the International Classification of Diseases (ICD) [8]. Each participant’s stroke severity score, measured using the National Institute of Health Stroke Scale (NIHSS) [9,10] and the Rankin Focussed Assessment Score [11] were collated as per hospital guidelines. The NIHSS is performed by a healthcare professional in the acute period following a stroke. Using 11 elements relating to neurological function, the NIHSS is scored between zero and 42, with 42 representing the most severe stroke. The NIHSS scores can be grouped according to stroke severity into minor (1–4), moderate (5–15), moderate to severe (16–20) and severe stroke (21–42). During follow up assessment at six months, participants then completed the 15 Patient Reported Outcome Measures (PROMs).

### 2.2. Scoring of PROMs

**Figure A1:**
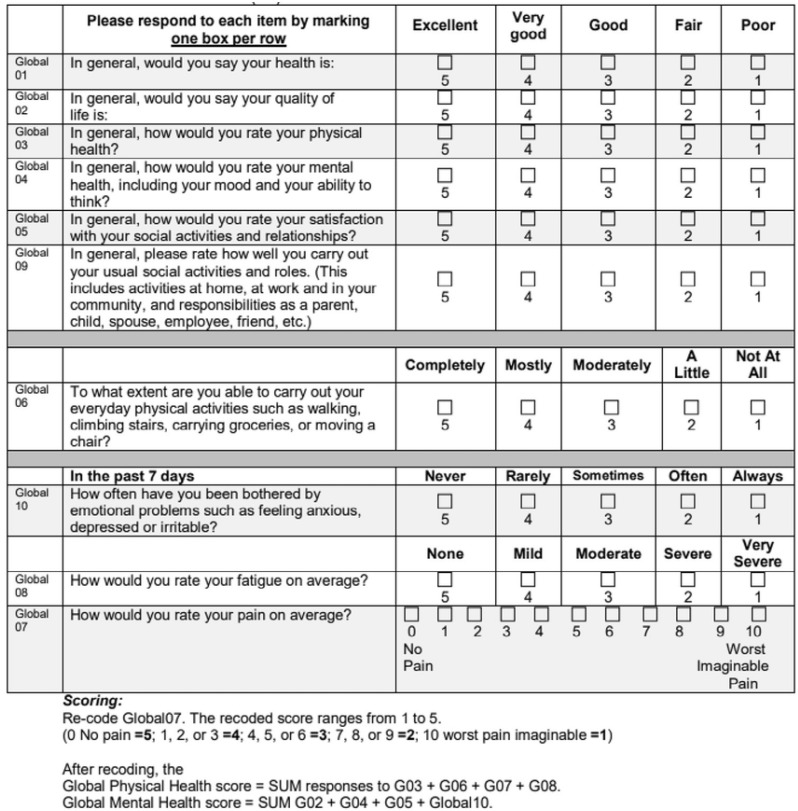

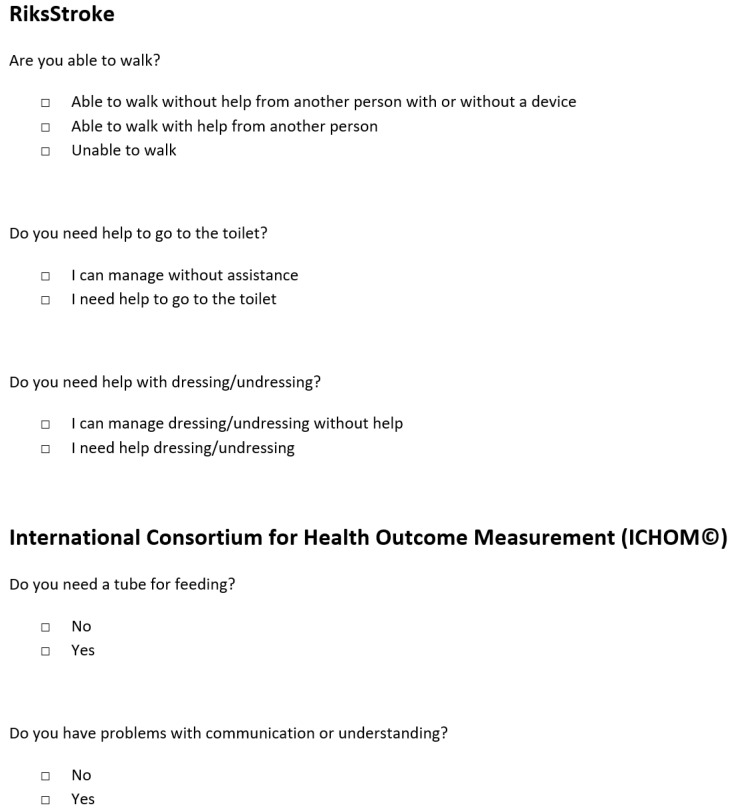
The 15 questions used in the PROM.

The fifteen Patient Reported Outcome Measures (PROMs) included ten questions from the PROMIS-10 v1.2 [12,13] and five additional stroke-specific questions. Three additional questions, relating to ambulation, toileting and dressing were implemented from the Riks-Stroke Questionnaire [14,15] and two questions from the ICHOM Stroke Standard Set were used to assess feeding and communication [4] (Figure A1).

The PROMIS-10 consists of ten equally weighted questions, with a mental and physical health domain, each with a total raw score between 4 and 20. Higher scores indicate better clinical outcomes and participant’s raw scores were converted to their equivalent t-score as defined by the Global Health Scoring Tool [16].

The additional five specific stroke questions (taken from the ICHOM and Riks-Stroke) consisted of both ordinal and dichotomous data. These were scored to give a total raw score for the stroke-specific domain of between four and ten, again with a higher score indicating a better outcome. These were scaled to give an equivalent t-score as for those in the mental and physical health domains of the PROMIS10 questionnaire.

Each question from PROMIS-10, Riks-Stroke and ICHOM was equally weighted, then summed and scaled to give a total maximum score out of 100.

### 2.3. Missing Data

Mean imputation was used within each PROM domain for participants with a completion rate of over 75% to predict the missing data. Participants who consented and completed the baseline characteristics but failed to complete the PROM were included in the followed-up population (*n* = 348), but not included in the PROM population used for the main analysis population (*n* = 343).

### 2.4. Statistical Analysis

An analysis of all participants was undertaken to describe the external validity of the population, comparing the participants that were followed up versus those that were not. A comparison of baseline clinical characteristics was completed. Linear regression of the NIHSS and the PROM was completed. A summary of the PROM domains (mental health, physical health and stroke-specific function) and total was conducted for each of the clinical characteristics.

We analysed the PROM total score in a crude and multivariable regression model, adjusted by participant age group, sex, classification of stroke and recruitment site. Adjusted mean differences (aMD) are presented with associated 95% confidence intervals calculations (95% CI), Stata version 15 was used.

## 3. Results

Participants were recruited between July 2017 and January 2018 across 14 centres within England and Wales. In total, 2074 participants were randomised to the trial, 458 participants (22.5%) were consented and 343 (16.3%) participants followed up. Those followed up were younger (mean age = 71.8 years [SD = 11.0]) and had a lower NIHSS score (mean = 5.3 [4.8]), whereas non-responders were older (mean age = 73.2 [13.6]), with a higher NIHSS score (mean = 6.3 [6.2]), demonstrated in Figure A1.

The demographic profiles of the participants who completed the PROM are provided in Table 1. There were 133 (38.8%) women, with a mean age of 71.7 years (30–94). Patient symptom severity was measured using the NIHSS, modified Rankin and PROMs. Using the NIHSS, 23 (6.9%) had no symptoms, 164 (47.8%) had mild symptoms, 132 (38.5%) had moderate symptoms and 17 (5.0%) had more severe symptoms. Using the modified Rankin, 92 people (33.3%) had no symptoms (mRS = 0), 80 (29.0%) were scored with no noticeable disability (mRS = 1) and 104 (30.3%) had more severe symptoms. Observation of PROM scores showed 56.8% of the severe cases were female, compared to just 28.8% of mild cases. Remaining clinical characteristics appeared to have little difference.

After the total PROM score was adjusted to predict the total NIHSS score, there was a strong association with the slope coefficient estimated as −0.10 (95% CI −0.14–−0.05; *p* < 0.001). Therefore, for an increase of 10 in the total PROM score, there was an estimated reduction in the NIHSS total score of 1 (95% CI 0.50–1.40).

Each of the three domains of the PROM were found to map to the clinical characteristics. Additionally, consistency was seen between patients with worse NIHSS, higher modified Rankin scores and lower total PROM scores. For example, patients that were independent exhibited a t-score mean of 64.55 (SD = 6.10) for the stroke-specific domain, compared to 53.56 (10.69) for those who were carer-assisted (Table 2). Similar findings were seen in the total PROM score when observing the difference between asymptomatic patients and those with severe symptoms in the NIHSS (Table 2).

### Statistical Analysis

Women reported a lower PROM when compared to men with a mean difference (MD) of −3.42 (95% CI −5.82–−1.53; *p* = 0.005; Table 3), and those completing the PROM aged 70 to 80 reported a higher PROM (MD = 4.70; 95% CI 0.98–8.42; *p* = 0.01) compared to those aged under 60. Adjustments were made for participant’s age group, sex, stroke classification and recruitment site in a multivariable linear model. We found a lower PROM with the adjusted MD (aMD) of 3.85 (95% CI −6.30–−1.41; *p* = 0.002; Table 3) for women. Similar findings were found in the crude analyses of the remaining two mediators, with aMD reduction of 3.88 (95% CI −0.61–7.37; *p* = 0.097) for haemorrhagic stroke classification. For age groups, the aMD for 70–79-year-olds had a higher PROM score compared to patients under 60 (aMD = 4.47, 95% CI 0.69–8.25; *p* = 0.002).

## 4. Discussion

Long-term medical outcomes are being increasingly reported across all medical specialities, including stroke, using PROMs to assess a condition and its symptom burden from a patient’s perspective. Within stroke as a subspecialty, several of these outcome measures exist [17,18], however, they were not felt to truly capture the long-term impact of stroke from a patient’s perspective. A new set of PROMs was developed in 2016 [4], consisting of 15 questions, which is being established and evaluated within the stroke community [6]. It was developed by consensus, involving a range of stroke specialists, stroke survivors and experts in outcome standard set development. These results constitute the largest use of this stroke-specific PROM to date, and therefore are likely representative of a UK stroke population. This study contributes to the evaluation process of this stroke-specific PROM, whilst confirming that strokes which are severe on admission, as demonstrated by NIHSS, also result in poorer PROMs at six months post-stroke. While these results are to some extent, to be expected, they will highlight to clinicians the likelihood of comorbidity, both physical and mental, which will facilitate earlier interventions and patient-centred discussion.

This study used randomly selected data acquired from a population which is representative of stroke survivors. This population was predominantly in the community following hospital discharge. It demonstrated that PROMs reported six-months following a stroke are correlated with the severity of stroke calculated on admission. Stroke severity is determined by a multitude of factors including the type of stroke and pre-hospital response time, as well as a patient’s perception of symptom severity. A rapid response is influenced by speed of first contact with emergency services, location in a rural or urban area and recognition of stroke symptoms (which itself is affected by patient education and those living alone) [19,20].

Interestingly, females with the equal NIHSS as males on admission reported worse symptoms at six months. This is supported by a review of sex differences within stroke which showed women have lower odds of a good outcome and higher prevalence of post-stroke depression [21]. Women report worse outcomes following acute illnesses [22,23,24], these differences are found to be significant even when adjusting for clinical variables such as age and pre-stroke health [21], although there is little understanding as to why these differences occur. Within stroke, women have been shown to experience aphasic disorders, visual field disturbances and dysphagia to a greater extent than men [25]. Overall, this increased morbidity following stroke in women is likely to contribute to worse patient reported outcomes measures [26].

Similar results were seen in patients suffering haemorrhagic strokes, who also had lower PROM scores. This would be in keeping with expectations, as haemorrhagic strokes are associated with poorer clinical outcomes across most existing outcome measures [27,28].

The reasons for a lack of association with increasing age, admission NIHSS score and PROM score is less clear. Typically, all stroke outcomes worsen with increasing age [29]. The statistical correlation was observed in people aged 70–79 years, the largest group within this study, which raises the possibility of a lack of power to detect any changes in the other groups. Thus, the findings relating to age should be interpreted with caution.

A large study that compared several commonly-used PROMs concluded they are necessary to provide additional information beyond the traditional clinician-reported measures such as NIHSS or the modified Rankin Scale [30]. In this study, NIHSS had the lowest percentage of improvement or worsening of any of the scores evaluated, suggesting PROMs with psychosocial domains more accurately reflect a patient’s recovery [30]. However, collection of this information may be restricted by stroke severity, with severely impaired patients being unable to complete PROMs. Whilst proxies could be used, psychosocial domains may be difficult to accurately assess.

This study does have some limitations, with the data representing a secondary analysis of an RCT. The stroke survivors consenting to the study were shown to have a better admission NIHSS than those who did not consent. This implies that our participants are fitter than the stroke population as a whole, which limits generalisability. Additionally, baseline patient demographics and clinical demographics (regarding stroke) were collected, however, no data was collected on patient comorbidity. Patients with other physical and mental health conditions are likely to score worse on the mental health and physical health domains of the PROM, which we could not account for, potentially skewing our data.

## 5. Conclusions

This study demonstrates that Patient Reported Outcome Measures collected at six-month follow up show association with severity of stroke on admission to hospital. These outcome measures were shown to be worse in women and those who had suffered a haemorrhagic stroke. This study shows that stroke-specific PROMs, developed by the stroke community itself, are useful for quantifying patient’s symptoms and should be advocated. A new, stroke-specific PROM, will help the stroke community better evaluate the long-term outcomes that actually matter to stroke survivors themselves.

## Figures and Tables

**Table 1:** Baseline demographics and descriptive analyses of all participants.

Number of Participants		Total 343
Grouped Age	<60 60–69 70–79 80>	47 (13.7%) 91 (26.5%) 121 (35.3%) 83 (24.2%)
Sex	Female Male	133 (38.8%) 210 (61.2%)
Carer Assisted	Yes	44 (12.8%)
NIHSS Grouped Frequencies	No Symptoms Mild Moderate Moderate-Severe Severe Missing	23 (6.9%) 164 (47.8%) 132 (38.5%) 14 (4.1%) 3 (0.9%) 7 (2.0%)
Thrombolysis Status	Received	69 (20.5%)
Rankin Classification	0 1 2 3 4 5 Missing	92 (33.3%) 80 (29.0%) 39 (14.1%) 43 (15.6%) 20 (7.2%) 2 (0.7%) 67 (24.3%)
Stroke Classification	Ischaemic Haemorrhagic Not Specific	304 (88.6%) 34 (9.9%) 5 (1.5%)

**Table 2:** Clinical characteristics of the PROM domain score mean (SD).

		PROMIS Mental Health	PROMIS Physical Health	Stroke Specific Domain	PROM Total
Grouped Age	<60 60–69 70–79 80–89 ≥90	41.10 (8.93) 45.30 (9.95) 46.82 (9.93) 45.27 (9.84) 41.26 (12.04)	40.97 (9.79) 42.59 (9.33) 43.75 (8.95) 41.063 (9.33) 39.46 (10.00)	63.52 (7.27) 63.48 (6.31) 64.37 (6.76) 61.90 (8.90) 51.15 (14.80)	71.78 (10.83) 74.62 (10.76) 76.48 (10.42) 73.23 (11.60) 67.02 (15.88)
Sex	Female Male	43.74 (9.90) 45.77 (9.95)	40.62 (8.77) 43.68 (9.44)	62.03 (8.92) 63.82 (6.90)	72.30 (11.45) 75.73 (10.68)
Carer Assisted	No Yes	46.25 (9.56) 36.35 (8.44)	43.58 (9.02) 35.20 (7.75)	64.55 (6.10) 53.56 (10.69)	76.21 (10.02) 62.10 (10.24)
NIHSS Grouped Frequencies	No Symptoms Mild Moderate Moderate-Severe Severe Missing	45.13 (10.52) 45.95 (9.63) 44.16 (10.00) 42.44 (13.66) 36.96 (8.27) 47.25 (6.03)	42.75 (7.75) 43.46 (8.92) 42.27 (9.94) 38.36 (9.71) 32.23 (3.87) 37.64 (5.45)	62.11 (9.28) 64.78 (5.96) 62.51 (7.90) 57.54 (10.54) 42.31 (8.52) 61.90 (9.91)	73.89 (11.36) 76.23 (9.88) 73.35 (11.64) 68.10 (15.54) 57.23 (5.98) 73.09 (5.79)
Thrombolysis Status	Not Received Received	44.82 (9.96) 45.47 (10.33)	42.47 (9.00) 43.15 (10.50)	63.23 (7.89) 63.19 (6.93)	74.42 (10.95) 74.78 (11.94)
Rankin Classification	0 1 2 3 4 5 Missing	46.20 (11.30) 45.68 (8.95) 44.35 (8.49) 42.16 (9.50) 42.60 (11.30) 33.3 (9.36) 45.89 (9.49)	44.26 (10.18) 42.44 (8.95) 40.57 (8.32) 39.87 (8.13) 41.42 (8.16) 37.15 (19.30) 43.49 (9.46)	64.68 (5.44) 64.11 (7.35) 62.62 (7.80) 61.72 (8.51) 59.91 (7.70) 51.90 (14.09) 62.50 (9.44)	76.42 (11.12) 75.10 (10.48) 72.62 (10.59) 70.88 (10.91) 71.28 (11.32) 65.07 (20.83) 75.31 (11.37)
Stroke Classification	Ischaemic Haemorrhagic Not Specified	45.18 (10.03) 43.35 (9.72) 43.94 (7.57)	42.64 (9.40) 41.47 (8.90) 40.32 (4.43)	63.43 (7.06) 60.54 (11.95) 62.28 (12.11)	74.68 (11.02) 72.26 (12.28) 72.19 (5.59)

**Table 3:** Clinical characteristics associated with the PROM total score.

		Crude Mean Difference (95% CI)	*p*-Value	Adjusted Mean Difference (95% CI)	*p*-Value
Grouped Age	<60 60–69 70–79 ≥80	Ref 2.84 (−1.04, 6.72) 4.70 (0.98, 8.42) 0.85 (−3.08, 4.79)	Ref 0.151 0.013 0.670	Ref 2.13 (−1.82, 6.09) 4.47 (0.69, 8.25) 0.39 (−3.62, 4.40)	Ref 0.290 0.020 0.849
Sex	Male Female	Ref −3.42 (−5.82, −1.03)	Ref 0.005	Ref −3.85 (−6.30, −1.41)	Ref 0.002
Stroke Classification	Ischaemic Haemorrhagic Other	Ref −2.42 (−6.37, 1.53) −2.49 (−12.33, 7.35)	Ref 0.228 0.619	Ref −3.38 (−7.37, 0.61) −4.05 (−14.15, 6.06)	Ref 0.097 0.431

## Data Availability

Not applicable.

## Appendix A

**Table A1:** Trial Cohort Demographics for Completers, Non-Completers and Non-Responders.

		Completed Follow-Up	Did Not Complete Follow-Up	Total
Number of Participants		348 (16.3%)	1726 (6.4%)	2074
Grouped Age	<60 60–69 70–79 80–89 ≥90	47 (13.5%) 93 (26.7%) 122 (35.1%) 77 (22.1%) 9 (2.6%)	317 (18.4%) 302 (17.5%) 492 (28.5%) 475 (27.5%) 140 (8.1%)	364 (47.6%) 395 (16.4%) 614 (21.1%) 552 (13.0%) 149 (2.0%)
Sex	Female	135 (38.8%)	797 (46.1%)	932 (44.9%)
NIHSS Grouped Frequencies	No Symptoms Mild Moderate Moderate-Severe Severe Missing	135 (38.8%) 23 (14.0%) 167 (19.2%) 133 (17.2%) 14 (15.2%) 4 (4.9%) 7	797 (46.1%) 141 (86.0%) 701 (80.8%) 639 (82.8%) 78 (84.8%) 77 (95.1%) 90	932 (44.9%) 164 (7.9%) 868 (41.9%) 772 (37.2%) 92 (4.4%) 81 (3.9%) 97
Thrombolysis Status	Received	69 (20.2%)	237 (14.2%)	306 (15.3%)
Modified Rankin Classification	0 1 2 3 4 5 Missing	92 (23.7%) 81 (22.3%) 40 (17.0%) 43 (13.8%) 20 (9.9%) 3 (2.8%) 69	296 (76.3%) 282 (77.7%) 195 (83.0%) 269 (86.2%) 182 (90.1%) 106 (97.2%) 396	388 (24.1) 363 (22.6%) 235 (14.6%) 312 (19.4%) 202 (12.6%) 109 (6.8%) 465
Stroke Classification	Ischaemic Haemorrhagic Not Specified	308 (16.7%) 35 (17.2%) 5 (17.2%)	1533 (83.6%) 169 (82.8%) 24 (82.8%)	1841 (88.8%) 204 (9.8%) 29 (1.4%)
